# Attenuation of cisplatin-induced acute kidney injury by sanguinarine: modulation of oxidative stress, inflammation, and cellular damage

**DOI:** 10.3389/fphar.2025.1567888

**Published:** 2025-04-02

**Authors:** Nur Elena Zaaba, Suhail Al-Salam, Sumaya Beegam, Ozaz Elzaki, Fatima Aldhaheri, Anas Nemmar, Badreldin H. Ali, Abderrahim Nemmar

**Affiliations:** ^1^ Department of Physiology, College of Medicine and Health Sciences, United Arab Emirates University, Al Ain, United Arab Emirates; ^2^ Department of Pathology, College of Medicine and Health Sciences, United Arab Emirates University, Al Ain, United Arab Emirates; ^3^ College of Medicine, Gulf Medical University, Ajman, United Arab Emirates; ^4^ Emeritus Professor, Department of Pharmacology and Clinical Pharmacy, College of Medicine and Health Science, Sultan Qaboos University, Muscat, Oman

**Keywords:** sanguinarine, kidney injury, inflammation, oxidative stress, apoptosis, mitochondrial dysfunction

## Abstract

**Introduction:**

Cisplatin (CP)-induced acute kidney injury (AKI) is a significant side effect of CP chemotherapy, driven by oxidative stress and inflammation. Sanguinarine (SANG), an alkaloid from the rhizomes of Sanguinaria canadensis and poppy-fumaria species, exhibits antioxidant and anti-inflammatory properties. This study examined SANG’s effect on CP-induced AKI in mice and its underlying mechanisms.

**Methods:**

Mice were orally administered 5 mg/kg SANG for 10 days. On the seventh day, they received a single intraperitoneal CP injection (20 mg/kg) and were sacrificed on the 11th day.

**Results:**

SANG significantly improved CP-induced decreases in body weight, water intake, urine volume, relative kidney weight, creatinine clearance, albumin-to-creatinine ratio, and plasma urea and creatinine levels. It also reduced elevated plasma neutrophil gelatinase-associated lipocalin, kidney injury molecule-1, cystatin C, and adiponectin levels, as well as renal markers of inflammation and oxidative stress induced by CP administration. SANG normalized kidney mitochondrial dysfunction, DNA damage, and apoptosis caused by CP. It also inhibited the CP-induced increase in the expression of phosphorylated nuclear factor-κB and autophagy markers in the kidney. Histological analysis showed that SANG reduced acute tubular necrosis and intraluminal protein accumulation due to CP.

**Discussion:**

In conclusion, SANG mitigated CP-induced AKI by reducing inflammation, oxidative stress, DNA damage, apoptosis, and autophagy. Pending more comprehensive pharmacological and toxicological assessments, SANG may be regarded as a potential therapeutic agent for mitigating CP-induced AKI.

## Introduction

Cisplatin (CP) nephrotoxicity is a major side effect, afflicting up to 30% of cancer patients receiving cisplatin chemotherapy (Tang et al., 2023; Pabla and Dong, 2008). Despite its nephrotoxic effect, cisplatin is still the go-to platinum-based anti-cancer drug, due to its efficacy in treating several cancers including head and neck, bladder, lung, ovarian, and testicular cancers. It is also effective against carcinomas, germ cell tumors, lymphomas, and sarcomas in some organs (Ali et al., 2020a). The nephrotoxic effects of CP can be attributed to its accumulation in the kidneys, as it is primarily excreted through them via glomerular filtration and tubular excretion (Tang et al., 2023). This subsequently triggers various damaging pathways, which results in acute kidney injury (AKI), marked by a rapid decline of renal function within days of CP administration (Tang et al., 2023). The long-term prognosis for patients undergoing multiple cycles of CP treatments is poor, with reports indicating a substantial incidence of patients developing chronic kidney disease (CKD), which, if not treated can lead to end-stage renal failure (Tang et al., 2023; Latcha et al., 2016; Zaaba et al., 2023).

The mechanisms that underlie CP-induced AKI are multifactorial. However, there is enough evidence to suggest that intracellular stresses including oxidative stress and inflammation are the main mechanisms involved (Pabla and Dong, 2008; Qi et al., 2023). Therefore, the most plausible strategy to mitigate CP-induced nephrotoxicity seems to be by reducing oxidative stress and inflammation using antioxidant and anti-inflammatory agents, including plant-derived compound (Tang et al., 2023; Zaaba et al., 2024). This can be done before or concomitantly with CP treatment. Moreover, previous studies have shown that treatment with potent antioxidant and anti-inflammatory agents such as bisabolol, as well as curcumin and melatonin in rodents, have shown promising outcomes in reducing CP nephrotoxicity (Ali et al., 2020a; Zaaba et al., 2022).

Sanguinarine (SANG), derived from the root extract of *Sanguinaria canadensis* and poppy-fumaria species is an alkaloid with an untapped potential and has previously demonstrated broad spectrum of biological activities (Wang et al., 2017; Zheng et al., 2022; Yu et al., 2020; Zhao et al., 2024; Li P. et al., 2021). Previous studies have shown that SANG is a potent antioxidant and exerts anti-inflammatory protection against neuropathic pain in rat model of chronic constriction injury (Li P. et al., 2021) and could protect against dextran sulfate sodium-induced ulcerative colitis in mice by regulating the nuclear factor erythroid 2-related factor 2 (Nrf2)/nuclear factor-κB (NF-κB) pathway (Zhao et al., 2024). Despite its well-documented antioxidant and anti-inflammatory effects, the nephroprotective effect of SANG on CP-induced AKI has yet to be investigated.

Therefore, since oxidative stress and inflammation are well-established factors in the etiology of CP-induced AKI (Pabla and Dong, 2008; Qi et al., 2023; McSweeney et al., 2021) and SANG has previously been shown to have strong antioxidant and anti-inflammatory capabilities, we thought that it would be useful to study the effect of SANG in CP-induced AKI, a study that, to our knowledge, has not been reported yet. Here, we aim to evaluate the potential salutary effects of SANG in ameliorating the CP-induced AKI in mice, by assessing various markers of oxidative stress, inflammation, mitochondrial dysfunction, DNA damage, apoptosis, and autophagy.

## Materials and methods

### Animals and treatment

Six to 8 weeks old BALB/c mice (Research Animal Facility, College of Medicine and Health Sciences, UAE University, Al Ain, United Arab Emirates) of both sexes weighing 25–30 g were housed in temperature-controlled (22°C ± 1°C) rooms with 12-h light-dark cycles (lights on at 6 00 a.m.). They were given unrestricted access to filtered drinking water and commercially available laboratory chow (National Feed and Flour and Marketing Co., Abu Dhabi, United Arab Emirates). AKI was induced in mice by administering 20 mg/kg CP intraperitoneally (IP). This dosage was selected based on prior reports demonstrating its effectiveness in inducing adverse physiological and biochemical changes associated with AKI in mice (Zaaba et al., 2022; Al Za’abi et al., 2021a). High-performance liquid chromatography grade SANG with ≥98% purity was purchased from Sigma Aldrich Co. (St. Louis, MO, United States). SANG was dissolved in 0.1% dimethyl sulfoxide (DMSO) in saline and was administered daily by oral gavage at a dose of 5 mg/kg for 10 consecutive days (Zaaba et al., 2022; Niu et al., 2012). The mice were randomly distributed into four equal groups (n = 8) and were treated as follows:

• Control–received vehicle (0.1% DMSO in saline, 10 mL/kg) orally for 10 days and saline (10 mL/kg) via IP injection on the seventh day of treatment.
• CP–received oral treatment as above and CP (20 mg/kg) via IP injection on the seventh day.
• SANG + saline–received SANG (5 mg/kg) orally for 10 days, and saline (10 mL/kg) via IP injection on the seventh day.
• SANG + CP–received SANG (5 mg/kg) orally for 10 days and CP (20 mg/kg) via IP injection on the seventh day.

The mice were placed individually in metabolic cages on the 10th day of the SANG treatment and were sacrificed 24 h later (i.e., 11th day). The 24-h urine volume and water intake were recorded, and the collected urine samples were stored at −80°C pending further analysis.

### Sample collection

The mice were weighed before the first treatment and then again immediately before sacrifice. They were then anesthetized with sodium pentobarbital (60 mg/kg) intraperitoneally, and blood from the inferior vena cava was collected into tubes with sodium citrate (4%). The tubes were then centrifuged at 900 × *g* at 4°C for 15 min to separate the plasma, and the latter were subsequently kept at −80°C for later use. Immediately after blood collection, the kidneys were excised and weighed. A small piece of the top part of the right kidney from each animal was taken and fixed in 10% formalin for histopathological analysis, whereas the remaining part of the right kidney was wrapped and dipped in liquid nitrogen and stored at −80°C for further analysis. The left kidneys were processed simultaneously for comet assay.

### Homogenization and protein quantification

The kidney tissues were homogenized using Precellys^®^ homogenizer from Bertin Instruments (Bretonneux, France). Briefly, the thawed kidney tissues were transferred into 2 mL microcentrifuge tubes along with ten 2.0 mm zirconia beads from Biospec (Bartlesville, OK, United States) and 900 µL of 1% protease inhibitor cocktail from Thermo Scientific (Rockford, IL, United States) in potassium chloride buffer, and homogenized for three times of three cycles of 10 s at 6,500 × *g*. Sequentially, the homogenates were centrifuged at 14,000 × *g* for 20 min and aliquoted into 3 tubes, then stored at −80°C pending further analysis.

The protein concentration in kidney homogenates was estimated using the Pierce™ Protein Assay Kit from Thermo Scientific (Rockford, IL, United States) according to the manufacturer’s protocol.

### Biochemical analysis of plasma and urine

The concentration of urea in plasma was determined using the Berthelot enzymatic method as outlined in the protocol from Linear Chemicals (Barcelona, Spain) (Fawcett and Scott, 1960). The concentration of creatinine in both plasma and urine was estimated by Jaffe’s reaction, according to the protocol detailed by Cayman Chemicals (Ann Arbor, MI, United States) (Ali et al., 2013). The albumin concentration in urine was measured using the same total protein estimation protocol described above (Hashemipour et al., 2012; Khatami et al., 2005).

### Markers of kidney injury and inflammation in plasma and kidney homogenates

The concentrations of kidney injury markers neutrophil gelatinase-associated lipocalin (NGAL), kidney injury molecule-1 (KIM-1), cystatin c, and adiponectin in plasma, as well as the concentrations of pro-inflammatory cytokines, tumor necrosis factor α (TNFα), interleukin (IL)-6, IL-1β and transforming growth factor β1 (TGF-β1) in kidney homogenates were measured using ELISA kits from R&D Systems (Minneapolis, MN, United States) according to the protocol provided by the manufacturer.

### Markers of lipid peroxidation and oxidative stress in kidney homogenates

The concentration of thiobarbituric acid reactive substances (TBARS) was spectrophotometrically measured using malondialdehyde (MDA) from Sigma Aldrich Co. (St. Louis, MO, United States) to create a standard curve (Nemmar et al., 2015). The levels of total nitric oxide (NO) and reduced glutathione (GSH) were measured as per the protocols from Sigma Aldrich Co. (St. Louis, MO, United States). The superoxide dismutase (SOD) activity on the other hand was measured using a kit from Cayman Chemicals (Ann Arbor, MI, United States). The 2′,7′-dichlorofluorescein diacetate assay was used to measure reactive oxygen species (ROS) and was performed according to the previously established protocol (LeBel et al., 1992; Hamadi et al., 2025).

### Mitochondrial extraction and assessment of the activities of mitochondrial complexes in kidney homogenates

The mitochondria were extracted from the kidney tissues using a differential centrifugal method as elaborated in previous studies (Zaaba et al., 2024; Wieckowski et al., 2009). Subsequently, the mitochondrial homogenates were subjected to analysis to evaluate the mitochondrial enzymatic activities of complexes I, II and III, and IV as per the protocol previously described (Zaaba et al., 2024; Spinazzi et al., 2012).

### DNA damage in kidney

Immediately after the extraction, the left kidneys were processed to evaluate the DNA damage following the standard protocol previously described for comet assay (Zaaba et al., 2022; Olive et al., 1994). Using the image analysis Axiovision 3.1 software by Carl Zeiss (White Plains, NY, United States), the extent of DNA damage was evaluated by measuring the length of the DNA migration, which includes the diameter of the nucleus and the length of migrated DNA.

### Western blot analysis of phosphorylated (Phospho)-NF-κB, beclin-1 and microtubule-associated protein 1 light chain 3 beta (LC3B) in kidney homogenates

Western blot analysis was used to determine the protein expressions of phospho- NF-κB, beclin-1, and LC3B. Proteins from the kidney homogenates were separated electrophoretically using 10% sodium dodecyl sulphate polyacrylamide gels. The proteins in the gels were then transferred onto polyvinylidene difluoride membranes using the Trans-Blot Turbo Transfer System from Bio-Rad (Hercules, CA, United States) for 30 min. Immediately after the transfer, the immunoblots were blocked with 5% non-fat milk for 1 h at room temperature and afterward incubated with 1:1000 dilution of mouse monoclonal phospho-NF-кB, beclin-1 and LC3B antibodies from Santa Cruz Biotechnology (Dallas, TX, United States) at 4°C overnight. The following morning, the blots were incubated for 2 h with 1:10,000 dilution of rabbit anti-mouse IgG horseradish-peroxidase-conjugated antibody from Abcam (Boston, MA, United States), and subsequently before visualization, they were incubated with SuperSignal™ West Pico PLUS chemiluminescent substrate from Thermo Scientific (Rockford, IL, United States) for 5 min. To measure the band density against the control, the blots were incubated with 1:10,000 dilution of mouse monoclonal β actin antibody from Santa Cruz Biotechnology (Dallas, TX, United States), and band density was measured using ImageJ, an image analyzing program developed by the National Institute of Health (Bethesda, MD, United States).

### Histopathology

The histopathological analysis performed here was as per the standard protocol done previously (Nemmar et al., 2010; Nemmar et al., 2017; Ramadi et al., 2016), where kidney tissues fixed in 10% formalin were grossed, processed, embedded in paraffin blocks, and then sliced into 5 µm thick sections using a microtome from Leica Biosystems (Nussloch, Germany). Next, the paraffin ribbon sections were placed onto slides and stained with hematoxylin and eosin. The stained sections were then evaluated and scored by a histopathologist under a light microscope. The scoring system was semi-quantitative, based on the percentage of acute renal tubular necrosis observed in the sections. A score of 0 was given to sections with normal kidney architecture and no necrosis, a score of 1 for less than 10% necrosis, a score of 2 for 10%–25% necrosis, a score of 3 for 26%–75% necrosis, and a score of 4 for more than 75% necrosis (Zaaba et al., 2022; Al Za’abi et al., 2021a).

### Statistics

All statistical analyses were conducted using GraphPad Prism Version 7 for Windows (GraphPad Software Inc., San Diego, CA, United States). To determine the distribution of the data, the Shapiro-Wilk test for normality was initially applied. For datasets that followed a normal distribution, a one-way analysis of variance (ANOVA 1) was performed, followed by Holm-Sidak’s post-hoc multiple comparisons test to identify significant differences between groups. In contrast, data that did not meet the assumption of normality (including water intake, relative kidney weight, urea levels, NGAL, KIM-1, TNFα, IL-1β, ROS, SOD, total NO, mitochondrial Complex IV activity, NF-kB, and beclin-1) were analyzed using the non-parametric Kruskal-Wallis test, followed by Dunn’s post-hoc multiple comparison test for pairwise group comparisons. The data presented in the figures are expressed as the mean ± standard error of the mean (SEM), and a p-value ≤0.05 was deemed statistically significant.

## Results

### Physiological and biochemical parameters

**FIGURE 1:**
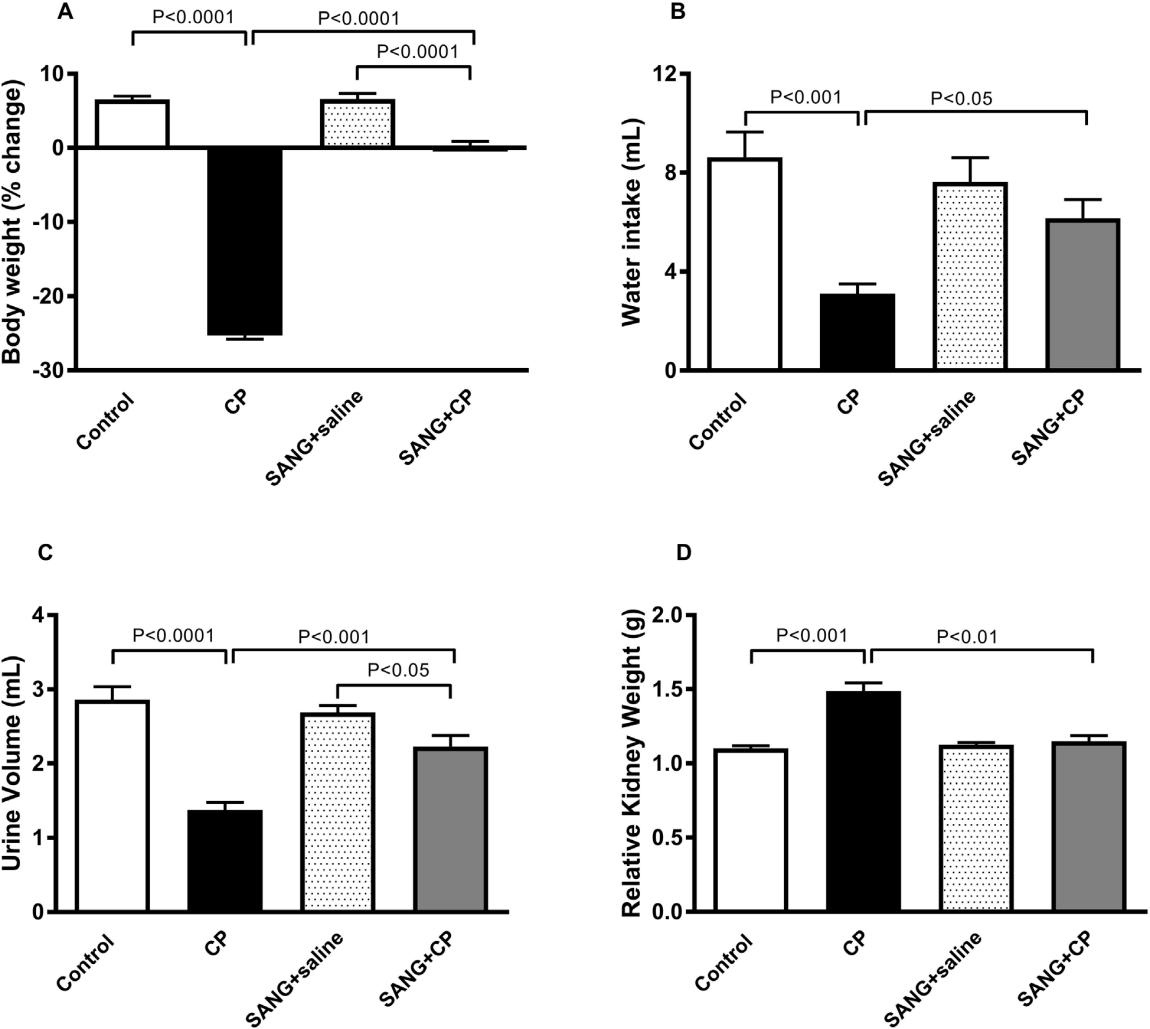
Body weight **(A)**, water intake **(B)**, urine volume **(C)**, and relative kidney weight **(D)** of mice treated orally with either vehicle (0.1% dimethyl sulfoxide in saline, control) or sanguinarine (SANG, 5 mg/kg) for 10 days, with or without cisplatin (CP, 20 mg/kg) given intraperitoneally on day 7 and sacrificed on day 11 (n = 8). Data was expressed as mean ± SEM.

As shown in Figure 1, the CP group displayed a marked reduction in body weight (*p* < 0.0001), water intake (*p* < 0.001), and urine volume (*p* < 0.0001), as well as a significant increase in relative kidney weight (*p* < 0.001), when compared with the control group. However, treatment with SANG has significantly ameliorated these effects (*p* < 0.0001, *p* < 0.05, *p* < 0.001, and *p* < 0.01, respectively).

**FIGURE 2:**
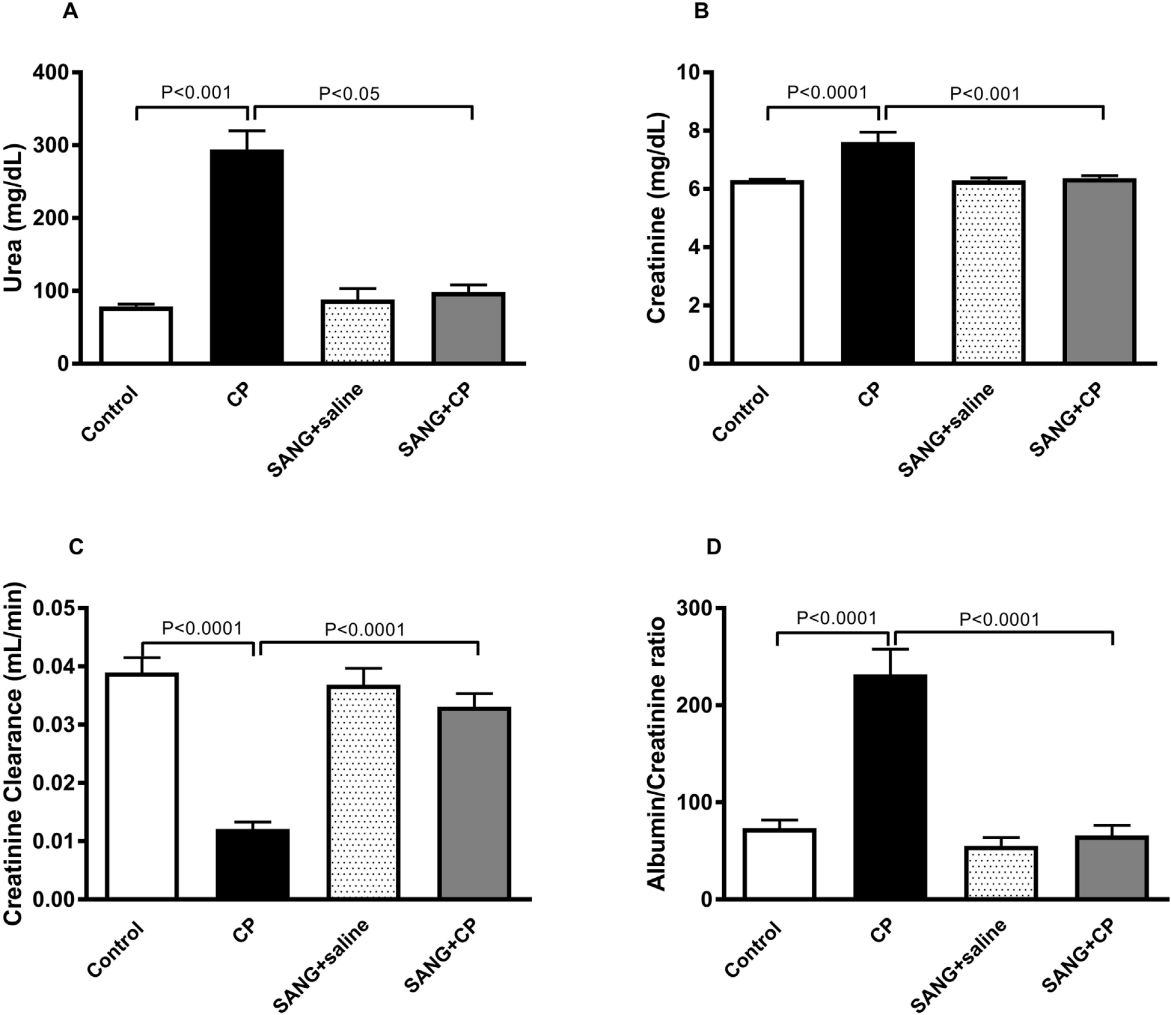
Urea **(A)** and creatinine **(B)** concentrations in plasma, as well as creatinine clearance **(C)** and albumin to creatinine ratio **(D)** of mice treated orally with either vehicle (0.1% dimethyl sulfoxide in saline, control) or sanguinarine (SANG, 5 mg/kg) for 10 days, with or without cisplatin (CP, 20 mg/kg) given intraperitoneally on day 7 and sacrificed on day 11 (n = 8). Data was expressed as mean ± SEM.

In Figure 2, the concentrations of urea (*p* < 0.001) and creatinine (*p* < 0.0001) in plasma showed significant elevation in groups treated with CP compared with the control. This elevation, however, was significantly abrogated when SANG was given alongside CP (*p* < 0.05 and *p* < 0.001). Mice treated with CP showed reduced kidney function, as evidenced by a significant reduction in creatinine clearance (*p* < 0.0001) compared with the control group. In addition, they also had a significant increase in albumin to creatinine ratio (*p* < 0.0001) as opposed to the control. The co-treatment with SANG, however, normalized these biochemical alterations (*p* < 0.0001 and *p* < 0.0001).

### NGAL, KIM-1, cystatin C, and adiponectin in plasma

**FIGURE 3:**
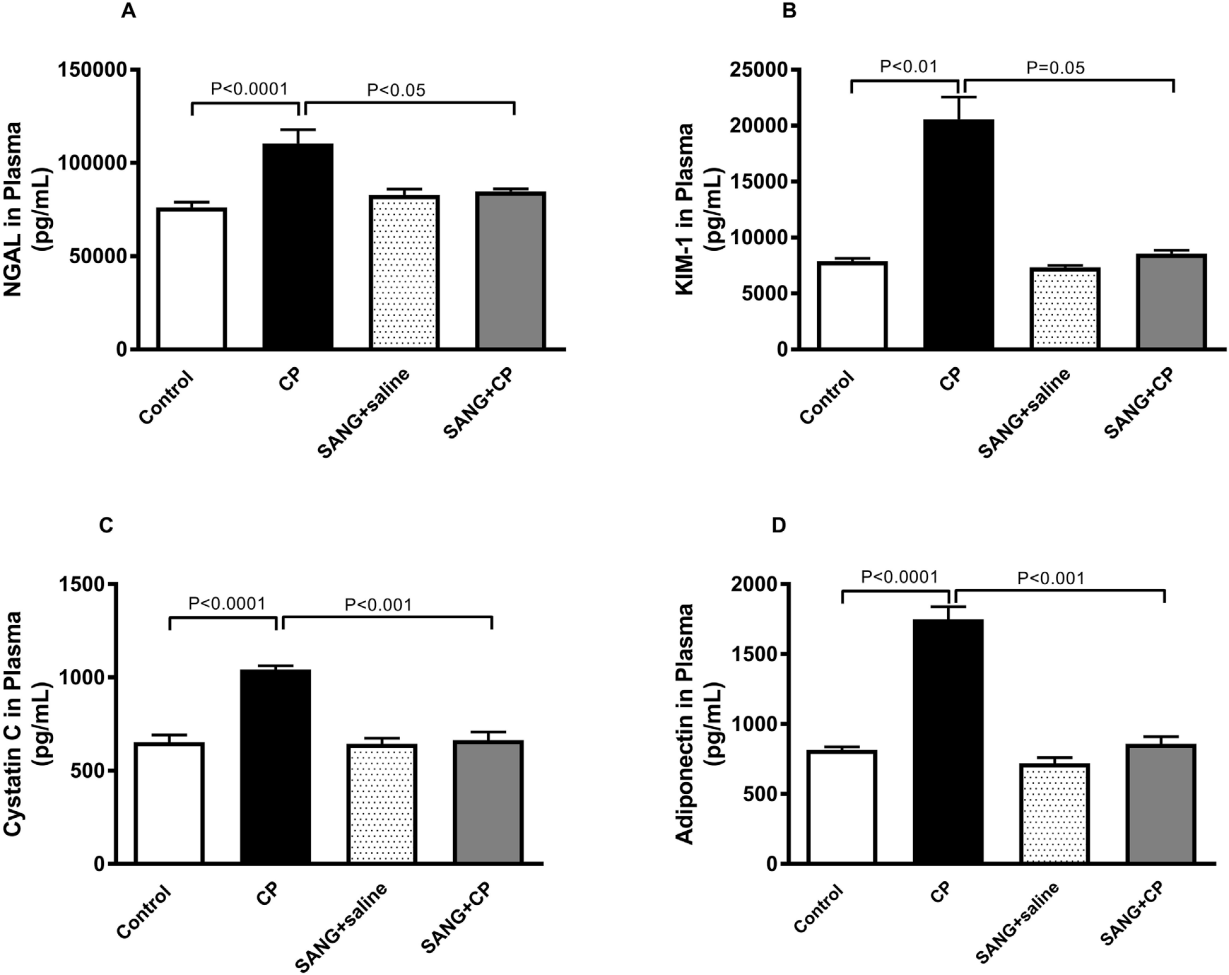
Neutrophil gelatinase-associated lipocalin (NGAL, **(A)**, kidney injury molecule-1 (KIM-1, **(B)**, cystatin c **(C)**, and adiponectin **(D)** concentrations in plasma of mice treated orally with either vehicle (0.1% dimethyl sulfoxide in saline, control) or sanguinarine (SANG, 5 mg/kg) for 10 days, with or without cisplatin (CP, 20 mg/kg) given intraperitoneally on day 7 and sacrificed on day 11 (n = 8). Data was expressed as mean ± SEM.

Figure 3 illustrates the changes in the levels of markers of kidney injury in the plasma across all four groups. The concentrations of NGAL (*p* < 0.0001), KIM-1 (*p* < 0.01), cystatin c (*p* < 0.0001), and adiponectin (*p* < 0.0001) were significantly increased when mice were given a single IP injection of 20 mg/kg CP. Nonetheless, when SANG was given concomitantly with CP, the plasma concentrations of these markers were significantly reduced (*p* < 0.05, *p* = 0.05, *p* < 0.001, and *p* < 0.001, respectively) when compared with the control group.

### TNFα, IL-6, IL-1β and TGF-β1 in kidney homogenates

**FIGURE 4:**
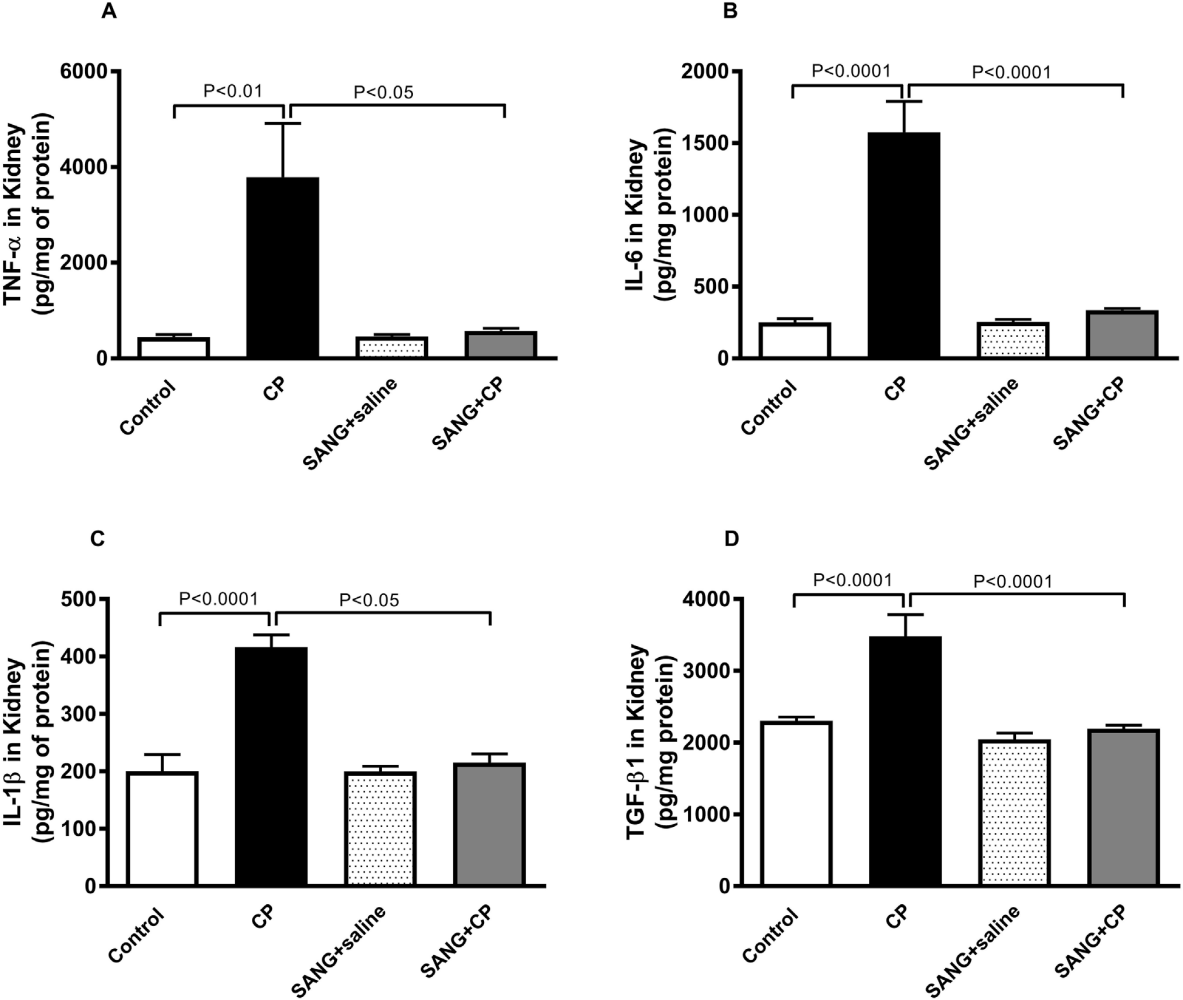
Tumor necrosis factor α (TNFα, **(A)**, interleukin (IL)-6 **(B)**, IL-1β **(C)**, and transforming growth factor β1 (TGF-β1, **(D)** concentrations in kidney homogenates of mice treated orally with either vehicle (0.1% dimethyl sulfoxide in saline, control) or sanguinarine (SANG, 5 mg/kg) for 10 days, with or without cisplatin (CP, 20 mg/kg) given intraperitoneally on day 7 and sacrificed on day 11 (n = 8). Data was expressed as mean ± SEM.

As shown in Figure 4, the concentrations of TNFα (*p* < 0.01), IL-6 (*p* < 0.0001), IL-1β (*p* < 0.0001), and TGF-β1 (*p* < 0.0001) in kidney homogenates were significantly augmented in CP-treated group compared with the control animals. These augmentations, however, were mitigated at a significant level when mice were co-treated orally with 5 mg/kg SANG (*p* < 0.05, *p* < 0.0001, *p* < 0.05, and *p* < 0.0001, respectively).

### TBARS, ROS, GSH, SOD, and NO in kidney homogenates

**FIGURE 5:**
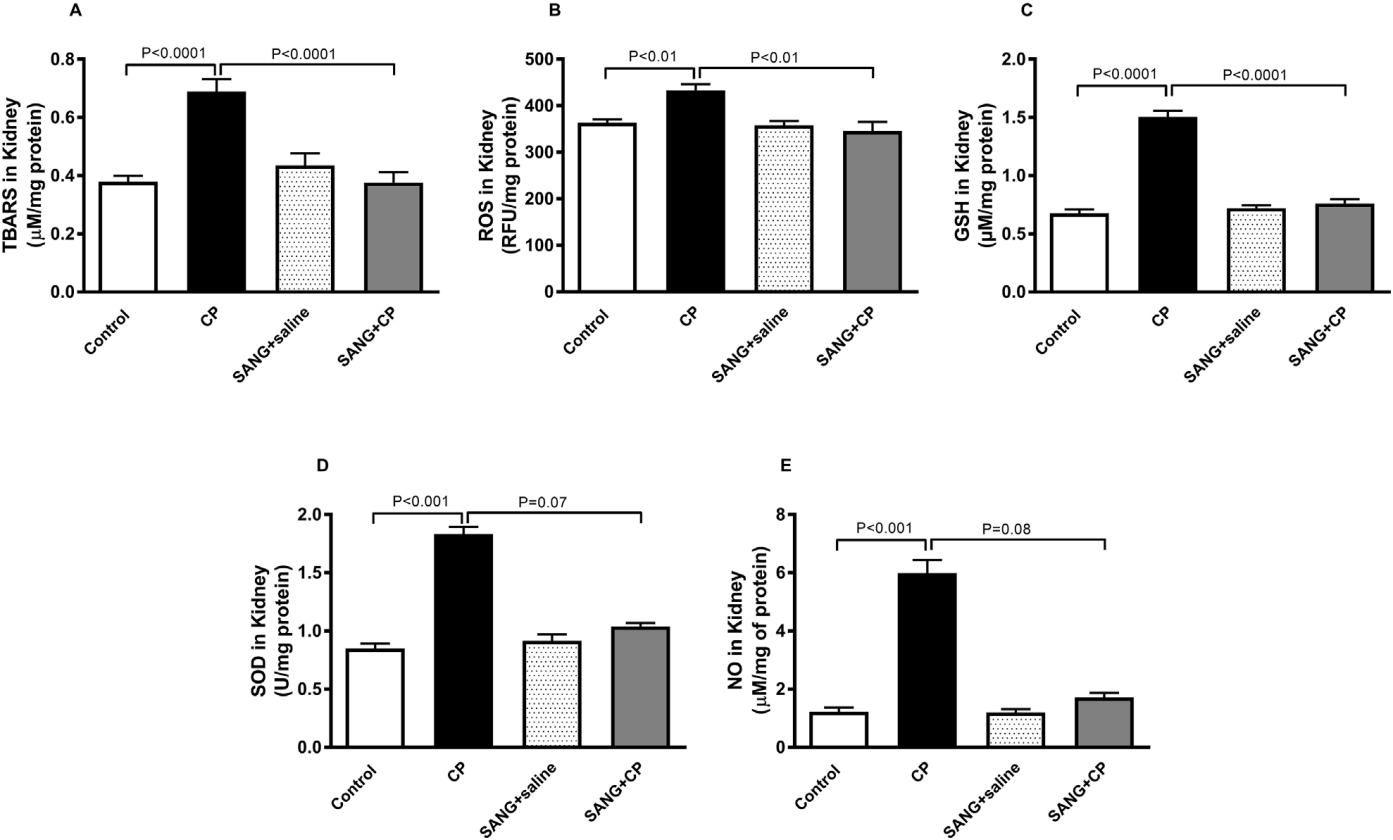
Thiobarbituric acid reactive substances (TBARS, **(A)**, reactive oxygen species (ROS, **(B)**, reduced glutathione (GSH, **(C)**, superoxide dismutase (SOD, **(D)**, and total nitric oxide (NO, **(E)** levels in kidney homogenates of mice treated orally with either vehicle (0.1% dimethyl sulfoxide in saline, control) or sanguinarine (SANG, 5 mg/kg) for 10 days, with or without cisplatin (CP, 20 mg/kg) given intraperitoneally on day 7 and sacrificed on day 11 (n = 8). Data was expressed as mean ± SEM.

Figure 5 depicts the levels of markers of oxidative stress in kidney homogenates. In the kidney homogenates of mice treated with CP, the level of TBARS was significantly elevated (*p* < 0.0001) and subsequently normalized when treated with SANG (*p* < 0.0001). Similar elevations were observed in the levels of ROS (*p* < 0.01), GSH (*p* < 0.0001), SOD (*p* < 0.001), and NO (*p* < 0.001) in the CP group when compared with the control. The elevation of ROS and GSH were markedly diminished with the pretreatment with SANG (*p* < 0.01 and p < 0.0001, respectively). The levels of SOD and NO were also decreased by SANG administration; however, this decrease did not reach statistical significance (*p* = 0.07 and *p* = 0.08, respectively).

### Mitochondrial complex I, complex II and III, and complex IV in kidney homogenates

**FIGURE 6:**
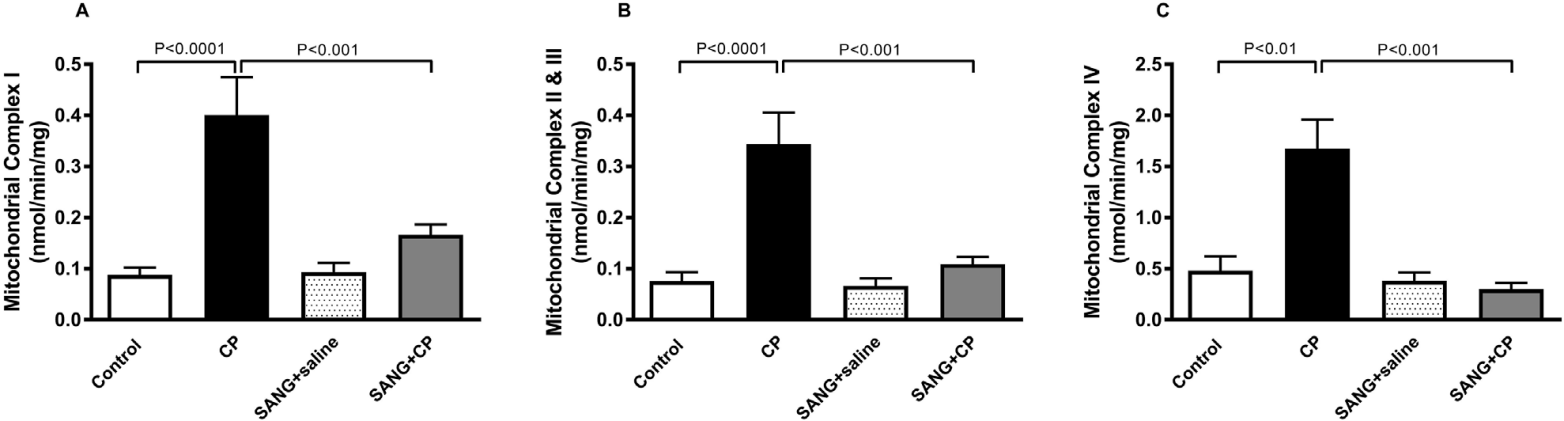
Mitochondrial complexes I **(A)**, II and III **(B)**, and IV **(C)** activities in kidney homogenates of mice treated orally with either vehicle (0.1% dimethyl sulfoxide in saline, control) or sanguinarine (SANG, 5 mg/kg) for 10 days, with or without cisplatin (CP, 20 mg/kg) given intraperitoneally on day 7 and sacrificed on day 11 (n = 8). Data was expressed as mean ± SEM.

Figure 6 highlights the mitochondrial activities in the kidney homogenates of all the groups. Mice treated with CP showed a significant increase in the activities of complex I (*p* < 0.0001), complex II and III (*p* < 0.0001), and complex IV (*p* < 0.01) compared with control mice. The increase in mitochondrial activities was significantly prevented with the treatment of SANG (*p* < 0.001, *p* < 0.001, and *p* < 0.001, respectively).

### DNA damage and cleaved caspase-3 in kidney homogenates

**FIGURE 7:**
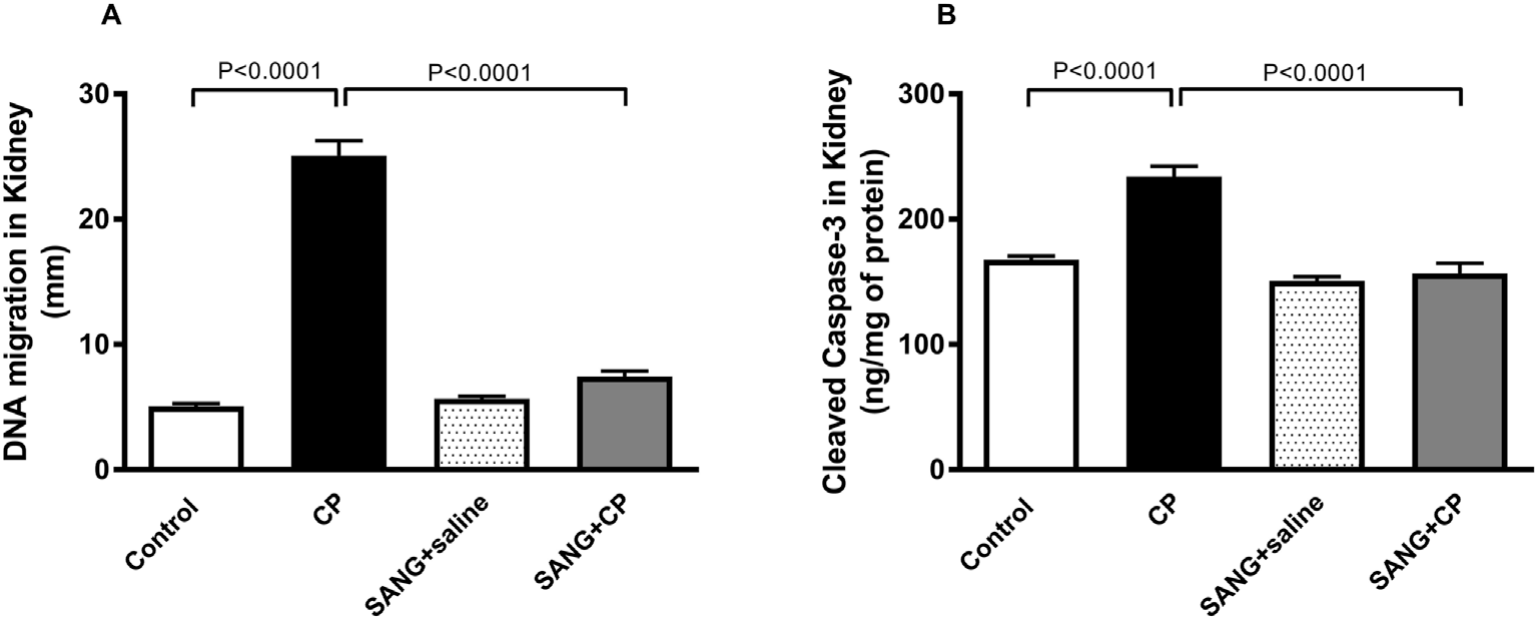
DNA migration (mm, **(A)** in kidney tissue by comet assay and the concentration of cleaved caspase-3 **(B)** in kidney homogenates of mice treated orally with either vehicle (0.1% dimethyl sulfoxide in saline, control) or sanguinarine (SANG, 5 mg/kg) for 10 days, with or without cisplatin (CP, 20 mg/kg) given intraperitoneally on day 7 and sacrificed on day 11 (n = 8). Data was expressed as mean ± SEM.

The level of DNA damage in the kidney of mice treated with CP was significantly higher compared with the control (*p* < 0.0001, Figure 7A) and this action was significantly reduced with the treatment of SANG (*p* < 0.0001). Similarly, CP treatment caused a significant increase in the concentration of cleaved caspase-3 (*p* < 0.0001, Figure 7B) in the kidney homogenates compared with the control group and SANG treatment has restored this increase (*p* < 0.000.1) significantly.

### Phospho-NF-κB, beclin-1 and LC3B in kidney homogenates

**FIGURE 8:**
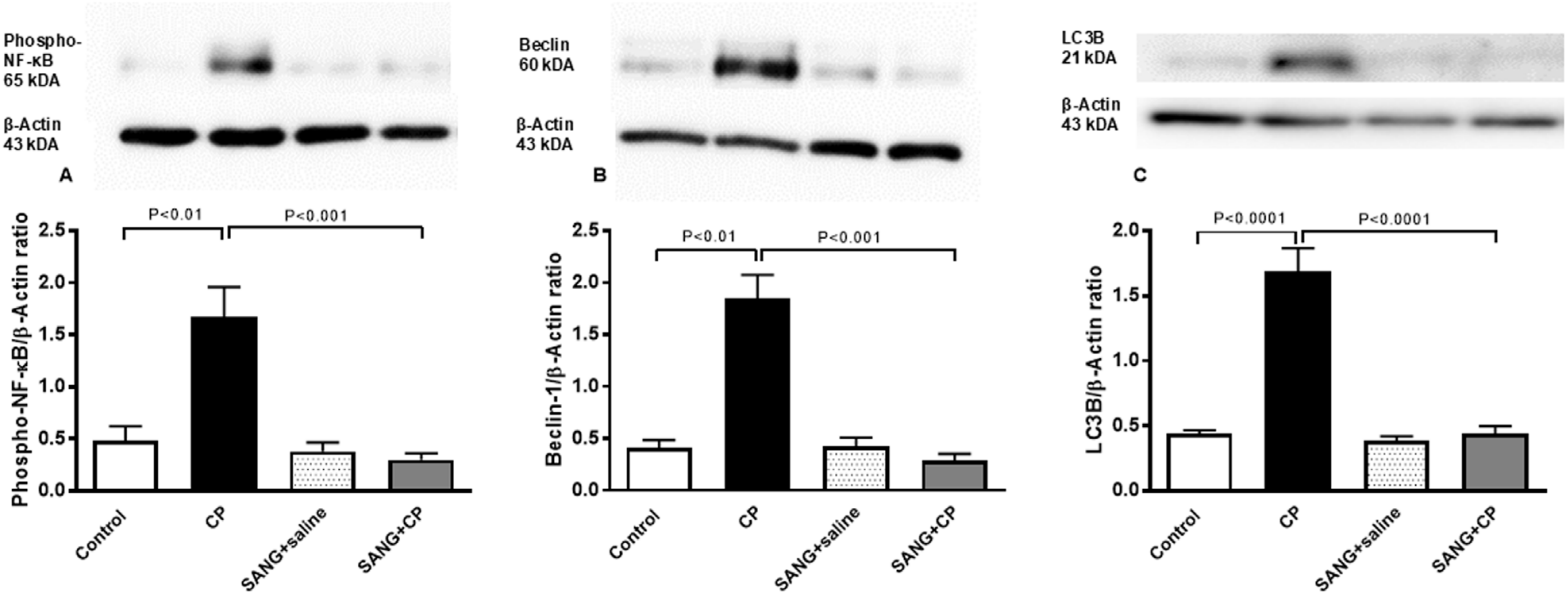
Phosphorylated nuclear factor-кB (phospho-NF-кB) **(A)**, beclin-1 **(B)**, and microtubule-associated protein 1 light chain 3 beta (LC3B, **(C)** expressions in kidney homogenates of mice treated orally with either vehicle (0.1% dimethyl sulfoxide in saline, control) or sanguinarine (SANG, 5 mg/kg) for 10 days, with or without cisplatin (CP, 20 mg/kg) given intraperitoneally on day 7 and sacrificed on day 11 (n = 8). Data was expressed as mean ± SEM.

Figure 8 illustrates that the protein expressions of phospho-NF-κB (*p* < 0.01), beclin-1 (*p* < 0.01), and LC3B (*p* < 0.0001) in kidney homogenates of mice treated with CP were significantly higher compared with the control mice. The treatment with SANG significantly reversed the overexpression of these proteins (*p* < 0.001, *p* < 0.001, and *p* < 0.0001, respectively).

### Kidney histology

**FIGURE 9:**
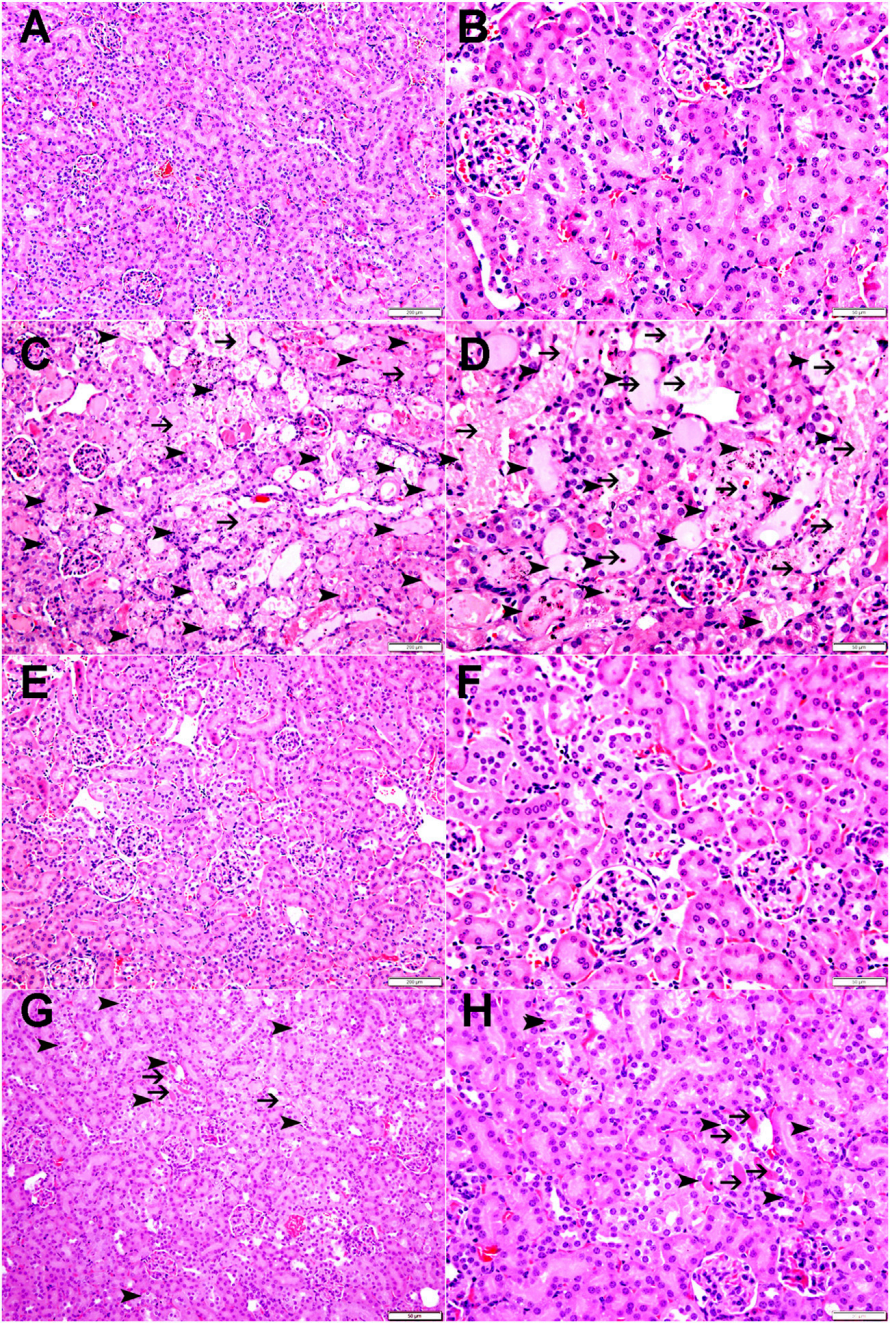
The representative images of light microscopy section of kidney tissue of mice treated orally with either vehicle (0.1% dimethyl sulfoxide in saline, control) or sanguinarine (SANG, 5 mg/kg) for 10 days, with or without cisplatin (CP, 20 mg/kg) given intraperitoneally on day 7 and sacrificed on day 11. The control group showed normal kidney architecture and scored 0 **(A, B)**. The CP-treated mice showed kidney tissue with large areas of acute tubular necrosis with tubular dilatation (arrowheads) in 68.66% of examined tissue areas with intraluminal protein secretions (thin arrows) in acutely injured tubules and were given a score of 3 **(C, D)**. The SANG + saline group showed normal kidney architecture and histology and scored 0 **(E, F)**. SANG + CP group showed kidney tissue with a dramatic decrease in areas affected by acute tubular necrosis (arrowheads) when compared with mice treated with CP and accounting for 27.5% of examined tissue areas with intraluminal protein secretions in a few acutely injured tubules (thin arrow) and scored 2 **(G, H)**.

As shown in Figure 9 and Table 1, the renal histopathological evaluation shows that the control group maintained a normal kidney architecture and scored 0. The CP-treated group, on the other hand, displayed acute tubular necrosis affecting 68.66% ± 3.8% of the examined kidney tissue areas with intraluminal protein accumulation, and was given a score of 3. The SANG group displayed normal kidney architecture similar to the control group, and therefore received a score of 0. Notably, the SANG + CP group showed significant improvement in renal tubules with 27.5% ± 1.8% of acute tubular necrosis and intraluminal secretion and therefore scored 2.

**TABLE 1:** Histopathological analysis and scoring of kidney sections of mice treated orally with either vehicle (0.1% dimethyl sulfoxide in saline, control) or sanguinarine (SANG, 5 mg/kg) with or without cisplatin (CP, 20 mg/kg) given intraperitoneally.

Group	% Of Necrotic area	Score of necrosis
Control	0 ± 0	0
CP	68.66 ± 3.8****	3
SANG + saline	0 ± 0°°°°	0
SANG + CP	27.5 ± 1.8^∆∆∆∆^	2

Data are expressed as mean ± SEM (n = 6). ****p < 0.0001 compared with the control group, °°°°*p* < 0.0001 compared with the SANG + CP group, and, ^∆∆∆∆^ *p* < 0.0001 compared with the CP group.

## Discussion

**FIGURE 10:**
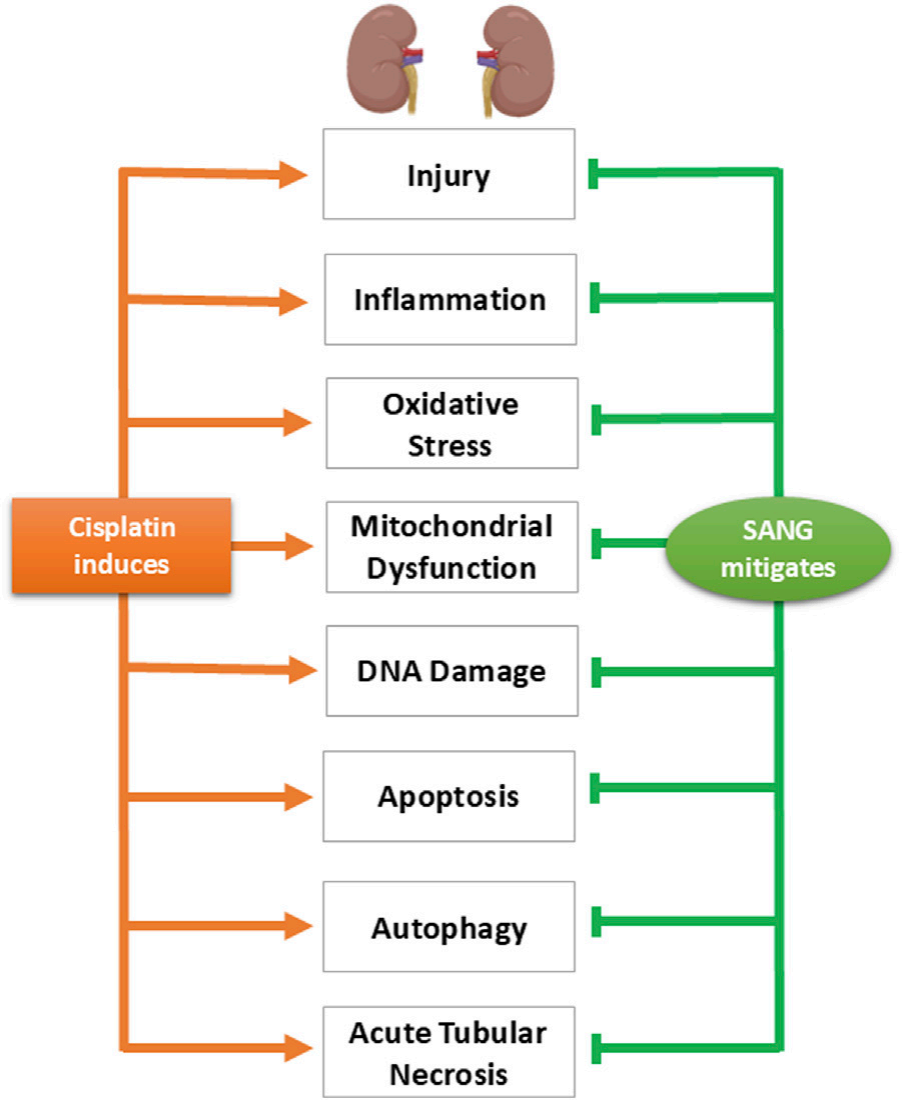
Diagram summarizing the key findings of the study, highlighting the physiological, biochemical, and histological alterations induced by cisplatin and illustrating the mitigating effects of sanguinarine (SANG) on these alterations.

The findings of this study showed that pretreatment with SANG substantially reduced the nephrotoxic effects induced by CP treatment. This mitigating effect occurred across various parameters associated with CP nephrotoxicity including markers of kidney injury, inflammation, oxidative stress, mitochondrial dysfunction, DNA damage, and apoptosis. Additionally, SANG has effectively restored the increased expression of phospho-NF-кB, a key regulator of inflammation, along with the autophagy markers beclin-1 and LC3B in the kidney, while also mitigating the structural damage caused by CP (Figure 10).

CP-induced AKI presents a major health concern for chemotherapy in cancer patients, with the global burden of AKI reported to be greater than that of breast cancer, heart disease, and diabetes (Kellum et al., 2021). The AKI cases in low-income countries are usually associated with community-acquired causes such as sepsis. However, in high-income countries, the majority of AKI cases are hospital-acquired, with diagnostic treatments including CP chemotherapy as one of its major contributing factors (Kellum et al., 2021). Although CP-induced AKI is reversible, repeated administration of CP over the course of chemotherapy may cause irreversible loss of nephron and precipitate the onset of CKD, which at this advanced stage, is irreversible (Kellum et al., 2021). Without interventions to reverse AKI or slow its progression to CKD, patients are likely to develop end-stage renal failure and become susceptible to cardiovascular complications, which increase their risk of premature death (Jankowski et al., 2021).

In efforts to mitigate the nephrotoxic effects of CP, various pharmacological, molecular, and genetic approaches have been attempted (Ali and Al Moundhri, 2006). This includes the use of antioxidants, diuretics, and cytoprotective agents (Ali and Al Moundhri, 2006; Zhou et al., 2022). Regardless of the protective attempts, the approach should not jeopardize the effectiveness of CP (Dewaeles et al., 2022) and should be easily available, effective, and economical. Natural products have been consistently preferred for some of these qualities (Zhou et al., 2022). In fact, 60%–75% of cancer and infectious disease drugs are derived from natural sources. (Boldi, 2004). Moreover, it has been reported that the co-administration of natural antioxidants and chemotherapeutic agents could potentially help reduce the toxic side effects of these agents (Zhou et al., 2022). In addition, combining strategies targeting multiple rather than a single pathway could provide a more robust nephroprotection. For example, agents that exhibit antioxidant properties could be administered together with agents that could inhibit DNA damage or apoptosis (Tang et al., 2023). SANG, a plant-based alkaloid that we have used in this study has been reported to possess all the aforementioned qualities (Zheng et al., 2022; Liu et al., 2015). This is one of the unique attributes of phytochemicals including SANG, where they have been shown to have multiple active targets, making it an attractive source for finding suitable therapy for diseases including CP nephrotoxicity (Shao et al., 2023). The dosage of CP used to induce AKI in our mouse model has been selected based on its efficacy in replicating the effects of CP-induced AKI in humans, and this model has been extensively utilized in the past due to its simplicity, reproducibility, and significant clinical relevance (Zaaba et al., 2022; Al Za’abi et al., 2021a; Al Za’abi et al., 2021b; Perše and Večerić-Haler, 2018). Moreover, it has been documented that in mice, clinical evidence of AKI nephrotoxicity develops 4–6 days after a single dose of CP injection, hence day 11 has been selected as our day of sacrifice, which corresponds to 4 days after CP injection, and this timeline aligns with the protocol that we have applied in our previous studies on CP-induced AKI in mice (Zaaba et al., 2022; Al Za’abi et al., 2021a; Perše and Večerić-Haler, 2018). In addition, we have employed a clinically applicable common route of precise oral dosing in experimental studies in rodents, the oral gavage, to administer 5 mg/kg of SANG, a dosage similar to that utilized in reports assessing its role in mitigating acute and chronic inflammation, as well as its antiparasitic effects in mice (Niu et al., 2012; Hoggatt et al., 2010; de Souza Silva et al., 2024). The treatment of SANG was initiated 6 days prior to CP injection and continued for a total duration of 10 days. The administration of SANG as a pretreatment was done in an effort to maximize the nephroprotective potential of SANG. This approach could be explored in clinical settings as a preventative strategy to reduce CP-induced nephrotoxicity before the start of chemotherapy.

In this study, the physiological changes observed are consistent with the experimental and clinical observations of AKI (Zaaba et al., 2022). The loss of body weight is a classic indicator of AKI where the body undergoes fluid loss and muscle wasting associated with catabolism, and loss of appetite due to gastrointestinal acidity (Bufarah et al., 2017; Ali et al., 2020b). The latter could also be linked to the reduction in water intake which subsequently leads to reduced urine output (Ali et al., 2020b). Whereas an increase in kidney weight ratio could indicate cellular hyperplasia induced by tubular injury (Zaaba et al., 2023; Humanes et al., 2017).

The treatment of CP has altered both traditional and novel biomarkers associated with kidney function in plasma and urine (Al Za’abi et al., 2021b). Our assessment of urea, creatinine, creatinine clearance, and albumin to creatinine ratio, the commonly measured kidney function parameters are in line with previously reported data on CP nephrotoxicity in rodents (Ali et al., 2020a; Al Za’abi et al., 2021b). Confirmatory data on the presence of CP-induced AKI were obtained through the measurement of augmented levels of relatively novel kidney injury markers NGAL, KIM-1, cystatin c, and adiponectin, which align with earlier findings (Ali et al., 2020a; Zaaba et al., 2022; Ali et al., 2020b). These alterations were restored to normal with the treatment of SANG.

Earlier studies have suggested that the etiology of AKI is associated with an expansive network of inflammatory responses. Particularly, TNFα has been identified as an important upstream regulator of CP-nephrotoxicity and mediates the expression of other cytokines (Pabla and Dong, 2008). It has been previously shown that CP treatment induces an increase in the concentrations of proinflammatory cytokines including TNF-α, IL-6 and IL-1β in the kidneys of mice and rats (El-Dawy et al., 2023; Zhang et al., 2023), which concurred with our observation in this study. Remarkably, the elevated level of TNFα, along with IL1-β and IL-6, and the profibrotic and pro-inflammatory cytokine TGF-β1 induced by CP were significantly reduced in mice treated with SANG. This observation is consistent with a previous study that highlighted the anti-inflammatory properties of SANG in mitigating acute and chronic inflammation in mice both *in vitro* using peritoneal macrophages and *in vivo* using various animal models of inflammation, including xylene-, formaldehyde-, and carrageenan-induced paw edema (Niu et al., 2012). Moreover, SANG has been reported to produce anti-inflammatory and neuroprotective actions following cerebral ischemia in animal model of middle cerebral artery occlusion (Wang et al., 2017). Efforts to mitigate nephrotoxicity by inhibiting TNFα have been explored, with strategies including treatment with pentoxifylline, a TNFα inhibitor (Tang et al., 2023; Ramesh and Reeves, 2002). Although targeting a specific protein might yield an immediate result, the long-term impact on overall health remains unclear. A meta-analysis has suggested that although highly effective, anti-TNFα therapy could elevate the risk of infections and malignancies (Li J. et al., 2021). Therefore, using natural products that demonstrate a broad range of inflammatory and strong antioxidant properties could be a safer and more practical approach (Fang et al., 2021).

Studies across experimental and clinical settings have recognized oxidative stress as an important mechanism involved in CP-nephrotoxicity, suggesting that antioxidants may be used against CP-induced renal injuries (Pabla and Dong, 2008). In this study, we found that SANG significantly reduced the elevated level of TBARS, the byproduct of lipid peroxidation, suggesting yet another ability of SANG in preventing the oxidation of lipids, which are the main component of cell membranes (Kopecka et al., 2020). This finding is in line with prior studies in which SANG has been reported to protect against indomethacin-induced small intestine injury in rats and against ulcerative colitis in mice by reducing the level of MDA (Zhao et al., 2024; Lin et al., 2022). Moreover, we found that the levels of ROS, GSH, and SOD, along with the free radical scavenger NO were significantly elevated following CP treatment. While SANG treatment has significantly decreased the levels of ROS and GSH, the decrease of SOD and NO induced by SANG did not reach statistical significance. Previous data have reported that the treatment with SANG could alleviate ulcerative colitis in mice by reducing the level of ROS and could inhibit oxidative stress in LPS-stimulated mastitis in mice (Zheng et al., 2022; Zhao et al., 2024). The latter study, along with another investigation assessing SANG’s efficacy in ameliorating fatty liver disease in mouse hepatocytes have reported that SANG could enhance the expression of Nrf2 and peroxisome proliferator-activated receptor γ (PPAR-γ) which improves the antioxidant activity (Zheng et al., 2022; Tian et al., 2021). This claim, however, will require further investigation in order to elucidate the involvement of the Nrf2/PPAR-γ pathway in the nephroprotective effects of SANG against CP-induced oxidative stress.

Our data show that CP caused mitochondrial dysfunction and that the treatment with SANG prevented this effect. It has been suggested that CP-induced oxidative stress disrupts the mitochondrial respiratory chain, causing mitochondrial dysfunction (Pabla and Dong, 2008). Mitochondrial dysfunction has been associated with an increased production of ROS and could exacerbate oxidative stress and inflammation, creating a chain reaction of detrimental effects driven by oxidative stress (Nemmar et al., 2023). A H9c2 cardiac cells study reported that treatment with SANG could restore ROS-mediated mitochondrial dysfunction by reducing oxidative stress (Liu et al., 2015), corroborating our current data.

Despite the wealth of knowledge on the involvement of oxidative stress in CP-induced AKI, little is known about the specific molecular target of ROS in the kidney. It has been postulated that ROS, due to its reactivity, could also disrupt other molecules such as DNA (Tang et al., 2023; Pravin et al., 2024). In the kidney cells, CP transforms into an aqueous form that has a high affinity for DNA. This leads to DNA crosslinks and subsequently damages the DNA (Tang et al., 2023). In this study, we found that the event of DNA damage was significantly greater in mice treated with CP compared with the control, as previously observed (Zaaba et al., 2022). This increase, however, was normalized with the treatment of SANG. The presence of DNA injury subsequently activates the DNA damage response, triggering a cascade of events such as cell cycle arrest, DNA repair, and cell death, which includes apoptosis (Tang et al., 2023; Wang, 2001). It has been previously reported that treatment with CP upregulated the pro-apoptotic markers, such as cleaved caspase 3 and cleaved caspase 9 in mice, and induced apoptosis in HK2 cells (Wang et al., 2014; Fan et al., 2020). Our data are in agreement with these findings, where we found a significant increase in the level of cleaved caspase 3 in CP-treated mice. This increase was significantly diminished with SANG treatment and is in line with previously reported data, where SANG demonstrated potential in inhibiting apoptosis induced by angiotensin II in cardiac cells (Liu et al., 2015). On the other hand, other studies have observed the contrary and found that SANG could actually promote apoptosis in cancer cells such as squamous cell carcinoma cells (Patil et al., 2024), and papillary thyroid cancer cells (Khan et al., 2020). These discrepancies may be due to differences in cell type, experimental conditions, or dosage, highlighting the context-dependent effects of SANG.

To further explore the involvement of inflammation in the nephroprotective effect of SANG, we evaluated the expression of phospho-NF-κB. The NF-κB is fundamental in the induction of CP-induced inflammation as it regulates the secretion of other inflammatory mediators including TNFα, IL-1β, and IL-6, which collectively contribute to the exacerbation of renal injury (Li et al., 2023). In this study, we found that the CP-induced augmented expression of phospho-NF-κB was significantly normalized when SANG was given concomitantly with CP, indicating that SANG could mitigate NF-κB activation. This observation aligns with previous reports where SANG inhibited osteoclast formation and bone resorption in primary mouse bone marrow monocytes/macrophage cells and attenuated neuropathic pain in rats by suppressing NF-κB activation (Yu et al., 2020; Li et al., 2013). A previous study has reported that SANG could mitigate LPS-induced inflammation in rat cardiomyocytes by inhibiting the toll-like receptor 4, leading to the downstream suppression of the NF-κB signaling pathway (Meng et al., 2018). It has also been reported that SANG could inhibit the activation the Wnt/β-catenin signaling, an important regulator of the inflammatory pathway, which subsequently reduced mastitis in mice (Zheng et al., 2022). Moreover, SANG has been shown to regulate M2-mediated angiogenesis through the Wnt/β-catenin pathway in lung cancer cells (Cui et al., 2022). These findings collectively suggest that SANG may be a promising candidate for further investigation to assess its involvement in modulating TLR4 and Wnt/β-catenin signaling in attenuating CP-induced inflammation.

Autophagy is a lysosomal pathway involved in maintaining cellular homeostasis. In the event of CP-induced cell death, including apoptosis, autophagy will take part in degrading and recycling cellular components to maintain the tissue structure and function (Shao et al., 2023; Tang et al., 2020). Relevantly, in the current study, in CP-induced mice, the expression of beclin-1 and LC3B, markers of autophagy, were significantly increased, and probably for the first time, we have shown that the increase of these markers was remarkedly and significantly reduced with SANG treatment. This observation is in line with a recent study that has identified SANG as a natural autophagy modulator that could suppress the progression of oral squamous cell carcinoma (Peng et al., 2025).

Previously SANG has been shown to induce toxicity effects *in vitro* on cardiomyocytes of Wistar rats, leading to cardiotoxicity, *in vivo* in zebrafish embryos and mouse blastocysts, inducing embryotoxicity and in mice, causing hepatotoxicity (Choy et al., 2008; Hu et al., 2005; Chan, 2011; Yang et al., 2021). These discrepancies between the latter studies and those reporting anti-apoptotic and anti-autophagic effects of SANG can be attributed to the difference in cell type, experimental conditions, or the dosage of SANG applied, underscoring how the outcomes of SANG treatment can vary depending on the specific context in which it is used. For instance, the dose used in the present study was 5 mg/kg given orally and showed no adverse effects, whereas in the study of Choy et al., the hepatotoxicity was reported at the dose of 10 mg/kg given intraperitoneally (Choy et al., 2008). Moreover, one toxicological review has reported that the acute oral LD_50_ of SANG is approximately 1,658 mg/kg in rats (Singh and Sharma, 2018), while at a lower dose of 5 mg/kg, presently and previously in pigs and rodents has shown to be safe and did not induce any toxicity (de Souza Silva et al., 2024; Galadari et al., 2017; Kosina et al., 2004; Zdarilova et al., 2008). Additionally, it is important to consider SANG’s bioavailability as it could affect its efficacy and toxicity (Stielow et al., 2023). In fact, little is known about the pharmacokinetics and pharmacodynamics of SANG (Huang et al., 2024). It has been reported that SANG has poor water solubility which significantly reduces its bioavailability, and only a few studies have been conducted to improve its bioavailability (Huang et al., 2024). In this context, one study has reported the transformation of SANG into solid lipid nanoparticles. The latter has increased the solubility and dissolution rate of SANG, which in turn increased its oral bioavailability (Huang et al., 2024; Li et al., 2014). Another study reported the encapsulation of SANG with water-soluble carboxylatopillar[6]arene generating a stable inclusion compound which increased its water solubility and improved its antibacterial activity (Huang et al., 2024; Ping et al., 2017). In this study, while we did not assess the bioavailability of SANG, our data showed that SANG given orally at 5 mg/kg over the period of 10 days was devoid of any side effects and effectively reduced CP-induced AKI. However further studies are warranted in order to investigate the bioavailability of SANG using different doses and routes of administration.

From the histopathological data obtained, we found that mice treated with CP showed classic CP-induced structural renal damage, characterized by a significant presence of acute tubular necrosis along with intraluminal protein accumulation (Yang et al., 2017; Meng et al., 2017). Remarkably, pretreatment with SANG significantly mitigated the structural damage induced by CP, highlighting its potential in preserving the structural integrity of the kidney amidst CP-induced inflammation and oxidative stress.

This study has some limitations. While SANG at the concentration of 5 mg/kg has demonstrated nephroprotective effects on CP-induced AKI in mice, further studies are required to evaluate its dose-response effects, along with its long-term safety and efficacy. Moreover, this study was conducted on healthy mice rather than on cancer-bearing models, which would have accurately mirrored clinical cases of CP nephrotoxicity. Additionally, although SANG has been tested in models of acute kidney injury, its effects in chronic kidney disease (CKD) remain unexplored. Hence, studying its efficacy in animal models of CKD would provide a valuable insight into its nephroprotective potential.

## Conclusion

Our findings demonstrated that SANG significantly mitigated CP-induced AKI in mice, potentially by reducing CP-induced inflammation and oxidative stress, as well as mitochondrial dysfunction, DNA damage, apoptosis, autophagy, and kidney structural damage.

## Data Availability

The original contributions presented in the study are included in the article/Supplementary Material, further inquiries can be directed to the corresponding author.
