# Supplementary material for: Attenuation of cisplatin-induced acute kidney injury by sanguinarine: modulation of oxidative stress, inflammation, and cellular damage
Source: Front Pharmacol. 2025 Apr 2;16:1567888. doi: 10.3389/fphar.2025.1567888 (PMC11999955; doi:10.3389/fphar.2025.1567888)
Supplement: Supplementary file 1 [file Presentation1.pptx]

## Slide 1
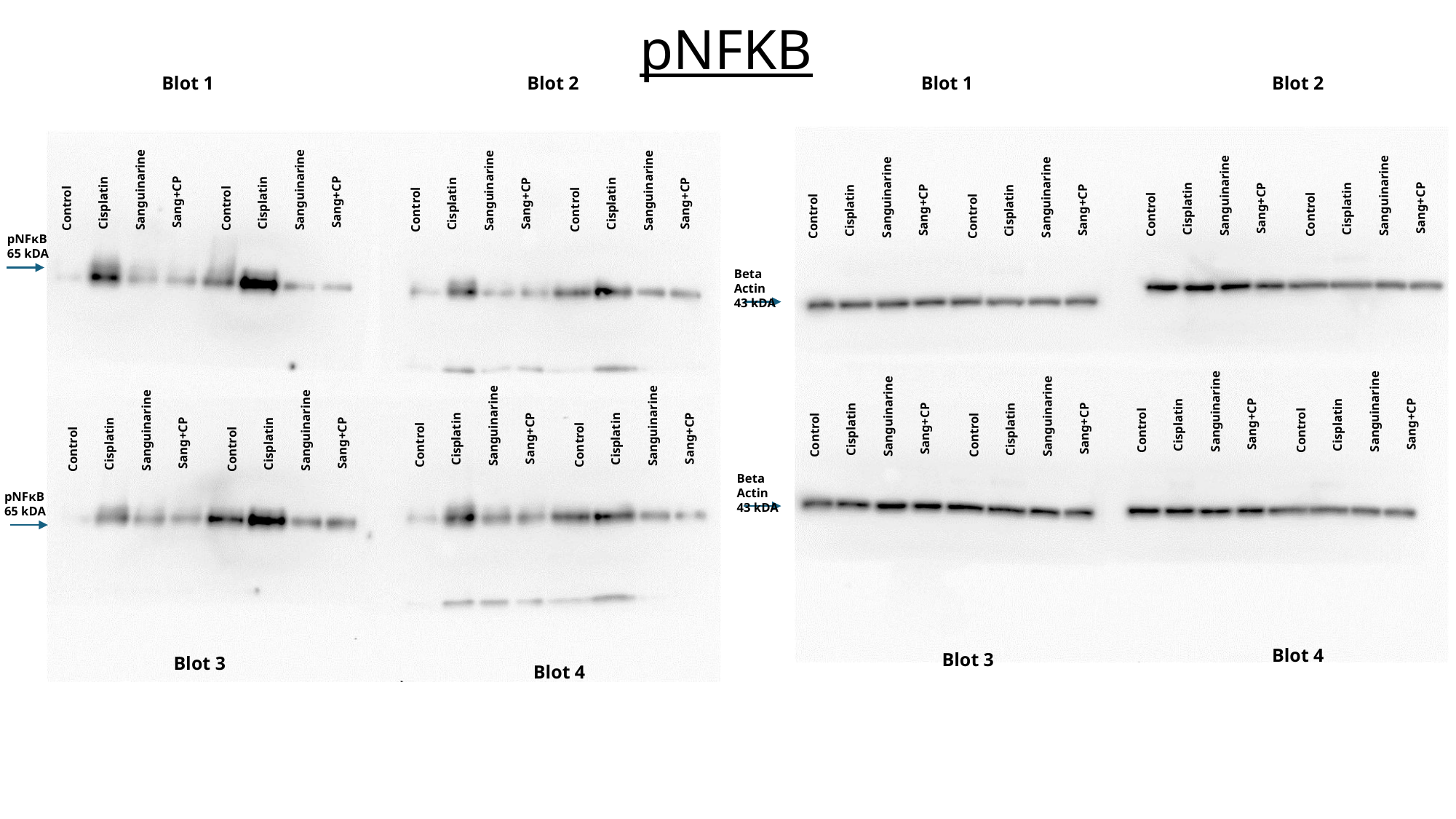

pNFKB
Blot 2
Blot 1
Blot 1
Blot 2
Sang+CP
Sang+CP
Sang+CP
Sang+CP
Cisplatin
Cisplatin
Sanguinarine
Sanguinarine
Cisplatin
Cisplatin
Sanguinarine
Sanguinarine
Sang+CP
Sang+CP
Cisplatin
Cisplatin
Sang+CP
Sang+CP
Sanguinarine
Sanguinarine
Cisplatin
Cisplatin
Sanguinarine
Sanguinarine
 Control
 Control
 Control
 Control
 Control
 Control
 Control
 Control
pNFκB
65 kDA
Beta Actin
43 kDA
Sang+CP
Sang+CP
Cisplatin
Cisplatin
Sanguinarine
Sanguinarine
Sang+CP
Sang+CP
Cisplatin
Cisplatin
Sanguinarine
Sanguinarine
Sang+CP
Sang+CP
 Control
 Control
Cisplatin
Cisplatin
Sanguinarine
Sanguinarine
Sang+CP
Sang+CP
 Control
 Control
Cisplatin
Cisplatin
Sanguinarine
Sanguinarine
 Control
 Control
 Control
 Control
Beta Actin
43 kDA
pNFκB
65 kDA
Blot 4
Blot 3
Blot 3
Blot 4

## Slide 2
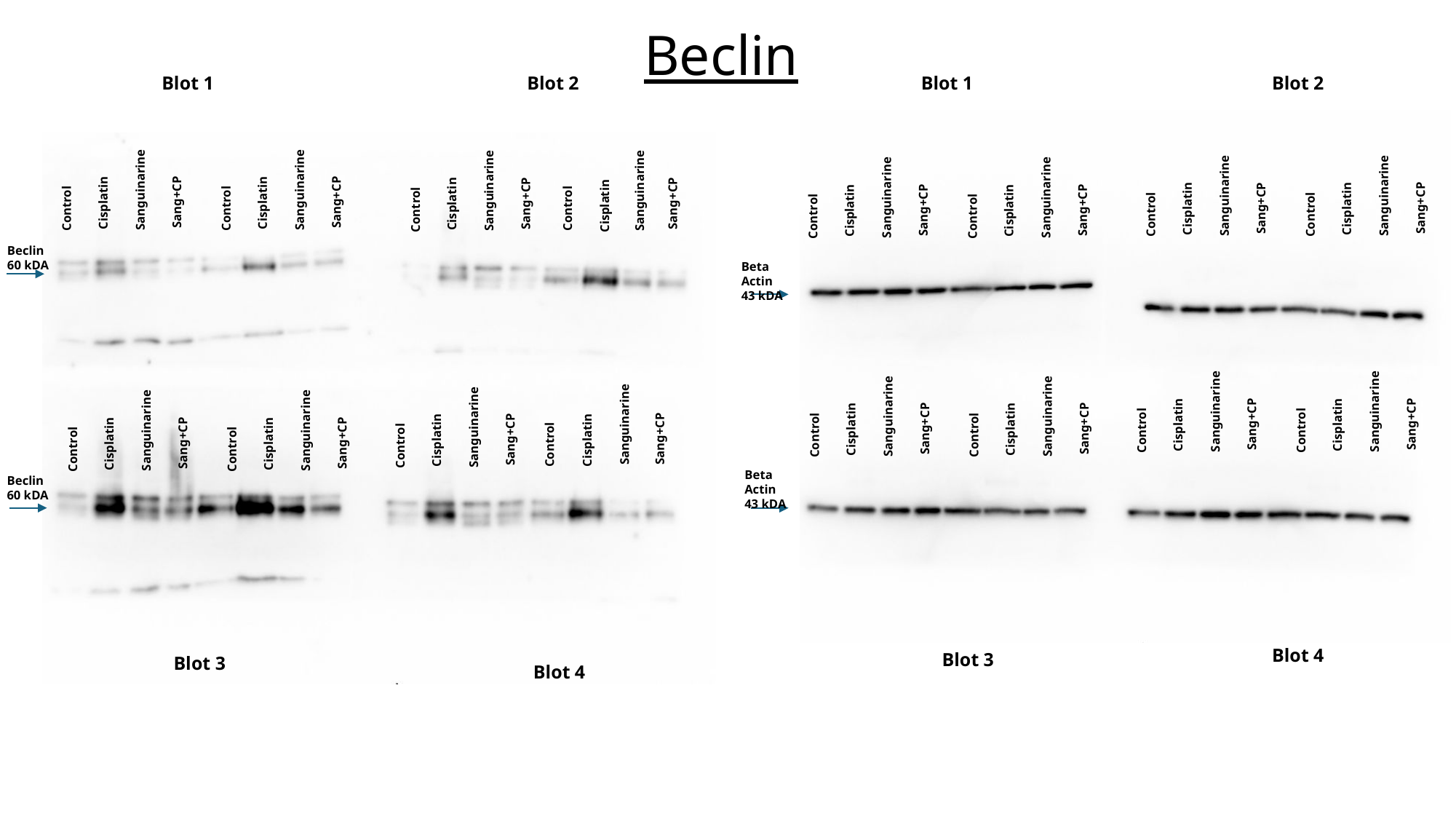

Beclin
Blot 2
Blot 1
Blot 1
Blot 2
Sang+CP
Sang+CP
Sang+CP
Sang+CP
Cisplatin
Cisplatin
Sanguinarine
Sanguinarine
Cisplatin
Sanguinarine
Sanguinarine
Cisplatin
Sang+CP
Sang+CP
Cisplatin
Cisplatin
Sang+CP
Sang+CP
Sanguinarine
Sanguinarine
Cisplatin
Cisplatin
Sanguinarine
Sanguinarine
 Control
 Control
 Control
 Control
 Control
 Control
 Control
 Control
Beclin
60 kDA
Beta Actin
43 kDA
Sang+CP
Sang+CP
Cisplatin
Cisplatin
Sanguinarine
Sanguinarine
Sang+CP
Sang+CP
Cisplatin
Cisplatin
Sanguinarine
Sanguinarine
Sang+CP
Sanguinarine
 Control
 Control
Sang+CP
Cisplatin
Cisplatin
Sanguinarine
Sang+CP
Sang+CP
 Control
 Control
Cisplatin
Cisplatin
Sanguinarine
Sanguinarine
 Control
 Control
 Control
 Control
Beta Actin
43 kDA
Beclin
60 kDA
Blot 4
Blot 3
Blot 3
Blot 4

## Slide 3
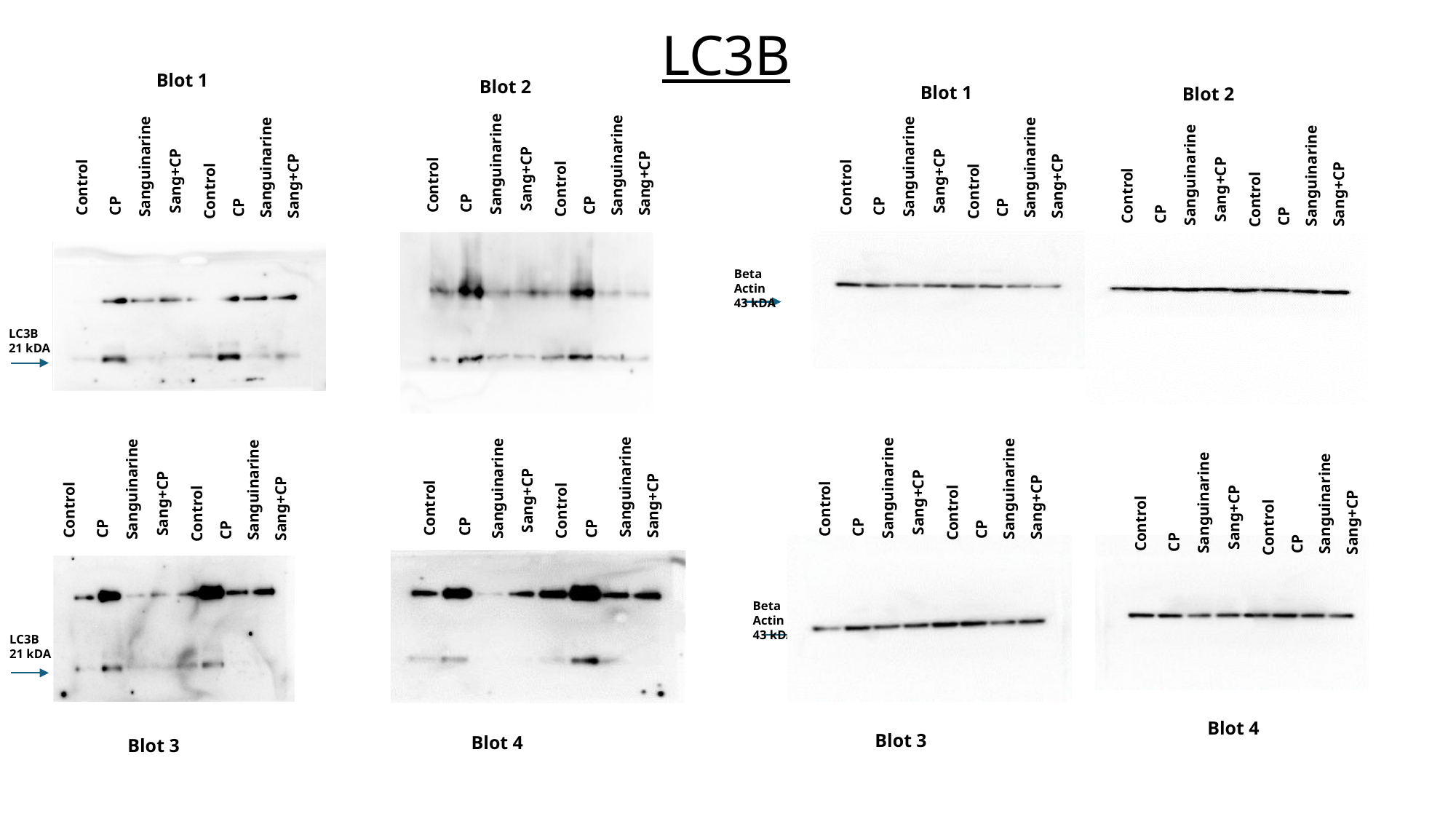

LC3B
Blot 1
Blot 2
Blot 1
Blot 2
Sang+CP
CP
Sang+CP
Sang+CP
CP
Sanguinarine
CP
CP
Sanguinarine
Sang+CP
CP
Sanguinarine
CP
Sanguinarine
Sanguinarine
Sanguinarine
Sang+CP
Sang+CP
Sang+CP
CP
CP
Sanguinarine
 Control
Sanguinarine
Sang+CP
 Control
 Control
 Control
 Control
 Control
 Control
 Control
Beta Actin
43 kDA
LC3B
21 kDA
Sang+CP
Sang+CP
Sang+CP
CP
CP
Sanguinarine
CP
Sang+CP
CP
CP
Sanguinarine
Sanguinarine
Sanguinarine
CP
Sanguinarine
Sang+CP
Sanguinarine
Sang+CP
 Control
Sang+CP
 Control
 Control
CP
 Control
CP
Sanguinarine
 Control
Sanguinarine
Sang+CP
 Control
 Control
 Control
Beta Actin
43 kDA
LC3B
21 kDA
Blot 4
Blot 3
Blot 4
Blot 3
